# Clinical Features and Surgical Management of Spinal Osteoblastoma: A Retrospective Study in 18 Cases

**DOI:** 10.1371/journal.pone.0074635

**Published:** 2013-09-18

**Authors:** Zhonghai Li, Yantao Zhao, Shuxun Hou, Ningfang Mao, Shunzhi Yu, Tiesheng Hou

**Affiliations:** 1 Department of Orthopaedics, First Affiliated Hospital of PLA General Hospital, Beijing, the People’s Republic of China; 2 Department of Orthopaedics, First Affiliated Hospital of PLA Second Military Medical University, Shanghai, the People’s Republic of China; University of Toronto, Canada

## Abstract

**Objectives:**

To investigate the clinical manifestation and surgical outcome of spinal osteoblastoma.

**Methods:**

From June 2006 to July 2011, 18 patients with spinal osteoblastoma treated surgically were analyzed retrospectively. There were 11 males and 7 females with an average age of 27.5 years（range, 16-38 years). The tumors were located at C5 in 7, C6 in 6, C7 in 3, C6-T1 1 in 1 and T11 in 1. Based on WBB classification, 16 were 1-3 or 10-12 and 2 were 4-9 and 1-3. 18 operations had been performed with en bloc resection. A posterior approach was used for 16 patients, and a combined posterior and anterior approach was used for 2 patients. Reconstruction using instrumentation and fusion was performed using spinal instrumentation in 13 patients. We used visual analogue scales (VAS) to evaluate the change of pain before and after the operation, and the McCormick System to assess functional status of the spine. Imaging test was used to review the stability and recurrence rate of spine cord, and the confluence of graft bones.

**Results:**

All cases were followed up for 24-80 months (average, 38.4 months). The average surgical time was 120.8 minutes (range, 80-220 minutes), with the average intraoperative blood loss of 520 ml (range, 300-1200 ml). During the follow-up period, the VAS grade reduced from 6.46±1.32 to 2.26±1.05 (*P* <0.05). 15 patients had neurological function improved and 3 remained no change which was evaluated by McCormick scale for spinal function status at final follow-up.

**Conclusions:**

Spinal osteoblastoma has its own specific radiographic features. There is some recurrence in simple curettage of tumor lesion. The thoroughly en bloc resection of tumor or spondylectomy, bone fusion and strong in Ter fixation are the key points for successful surgical treatment.

## Introduction

Osteoblastoma has been known by several different names, including benign osteoblastoma, giant osteoid osteoma and osteogenic fibroma. Since these different designations are not very suitable for distinguishing the type of tumor itself, the name osteoblastoma is most commonly used and has become the generally accepted term [1]. Osteoblastoma is a generally benign or locally aggressive osteoblastic tumor that forms osteoid tissue and bone. Osteoblastoma is a rare form of bone tumor, which accounts for approximately 1% of all primary bone tumors and about 3% of benign bone tumors. The tumor occurs most frequently in the vertebral column and the long bones, followed by the bones of the hands and feet. Spinal lesions are usually present in the posterior elements and the vertebral arches. Although the vertebral bodies can be involved, it is extremely rare to find the tumor primarily located in this area [2-5]. Based on previous literatures review, spinal osteoblastoma does not exhibit characteristic clinical manifestations and the symptoms are often neglected during the early stages of the disease, which delays an accurate diagnosis and treatment [2-9].

In current study, a total of 18 patients with spinal osteoblastoma were admitted to our hospital and treated in our department between June 2006 and July 2011. We made a retrospective study on these cases with more than 3 years’ follow-up, aiming to compare these results with those reported in the literature to investigate the clinical manifestation and surgical outcome of spinal osteoblastoma. Weinstein- Boriani- Biagini (WBB) staging system was used as a guide to select the appropriate surgical path and determine the scope of the operation. All of these patients underwent surgery with satisfactory results.

## Data and Methods

### Clinical data

This study was approved by the ethics statement of our hospital (First Affiliated Hospital of PLA General Hospital, Beijing). Out of the 18 patients enrolled in this study, 11 were males and 7 were females, with an average age of 27.5 years (range: 16-38 years). Informed consent from the next of kin, caretakers, or guardians on the behalf of the minors/children participants involved in our study were obtained. The duration from the onset of symptoms to diagnosis of the disease was from 10 days to 6 years, with an average length of 15 months. The locations of the tumors included C5 (7 patients), C6 (6 patients), C7 (3 patients), C6-T1 (1 patients) and T11 (1 patients). The lesions were predominantly present in the transverse processes, spinous processes, laminae and pedicles, with vertebral body involvement occurring in 2 patients. According to the WBB staging system for primary spine tumors [10], the tumors were mainly present in zones 1-3 or 10-12, although zones 4-9 were involved in 2 patients. Intraoperative frozen sections and postoperative histopathology confirmed the diagnosis of osteoblastoma for all patients.

The earliest symptom in most patients was nonspecific pain, which gradually developed into nerve root symptoms or spinal cord compression. The main signs and symptoms that were observed in this study were as follows: 1) Pain: All patients complained of various intensities of posterior neck pain or back pain, and some also suffered nocturnal pain. Ten patients experienced nerve root pain, which radiated to the upper arm, while a few patients complained of soreness at the site of the lesion that involved vertebral segments. 2) Sensory disorder: Seven patients (38.9%) experienced numbness and itching with segmental skin hyperaesthesia or hypoaesthesia. 3) Dyskinesia: Eight patients (44.4%) suffered from different intensities of dyskinesia below the affected segments. 4) Sphincter dysfunction: Two patients (11.1%) had urination and defecation disorders, including urine retention, urinary incontinence and chronic constipation.

All patients underwent careful physical examination before surgery and focal neurological signs were recorded. Anteroposterior (AP) and lateral X-rays, computed tomography (CT) scans and magnetic resonance imaging (MRI) were routinely performed for these patients. The imaging test results were consistent with the focal neurological signs. Through these examination combined with clinical manifestations in patients, we can determine the location of the tumor primarily. If the results suggested possible involvement of the vertebral artery foramen by tumor, the patient was subjected to the examination of magnetic resonance angiography (MRA) to determine whether the vertebral artery was involved. Preoperative MRA of the cervical arteries was carried out in 6 patients. The results revealed vertebral artery compression and thinning on the affected side in 4 patients and compensatory enlargement of the vertebral artery on the unaffected side occurred in the remaining 2 patients.

### Surgical methods

The surgical approaches and spinal reconstruction methods were chosen based on the extent of vertebral involvement combined with the stage of the tumor, as classified according to the WBB staging system for spine tumors. Sixteen patients were subjected to tumor excision through a posterior approach and 2 patients underwent tumor excision through a combined single-stage anterior and posterior approach. Spinal reconstruction was selectively performed based on the extent of lamina resection, whether the pedicle was removed and whether the facet joint injury occurred. In this study, there were 13 patients with the lesion involving facet joints, vertebral pedicles and vertebral bodies. These patients received spinal reconstruction to restore spinal stability after the excision of the tumor. Among them, 11 underwent posterior vertebral pedicle or lateral mass screw fixation, and two cases had anterior cervical plate fixation and spinal fusion using a bone graft. Specific steps were taken to make the instrumentation stage of this procedure safer for four patients with VA compression and compensatory contralateral enlargement. These patients received spinal reconstruction to restore spinal stability after the excision of the tumor. Among them, three cases underwent unilateral fixation on the unaffected side, and one case had fixation across the affected side to avoid the interference of vertebral artery at the lesion level. An intraoperative somatosensory evoked potentials (SSEP) was routinely performed, and a postoperative cervical collar was required to be worn for 3 months.

### Postoperative observation and follow-up

The follow-up examinations were scheduled at 3 months, 6 months and 1 year after surgery, and every half year beyond the first year after surgery. Imaging tests including X-rays, MRI and CT scans of the cervical spine were performed to determine whether tumor recurrence occurred, whether the spinal stability of the affected segments was restored, whether there was implant migration after internal fixation, and whether spinal fusion was achieved. Spinal fusion criteria included the following: 1) trabecular bone formation linking the bone graft and the adjacent spinal elements; 2) absence of lucencies around the bone graft; 3) absence of graft migration in dynamic plain radiographs. A Visual Analogue Scale (VAS) was adopted for the assessment of pain before and after surgery. Spinal cord function was assessed according to McCormick classification [11]. The therapeutic outcomes were described as improved, no change, or worse based on the preoperative and final follow-up McCormick grades of spinal cord function.

### Statistical analysis

The statistical software package SPSS 12.0 was used for statistical analysis, and a paired t-test was adopted to compare preoperative and final follow-up VAS scores. A *p* value of <0.05 was considered statistically significant.

## Results

**Figure 1:**
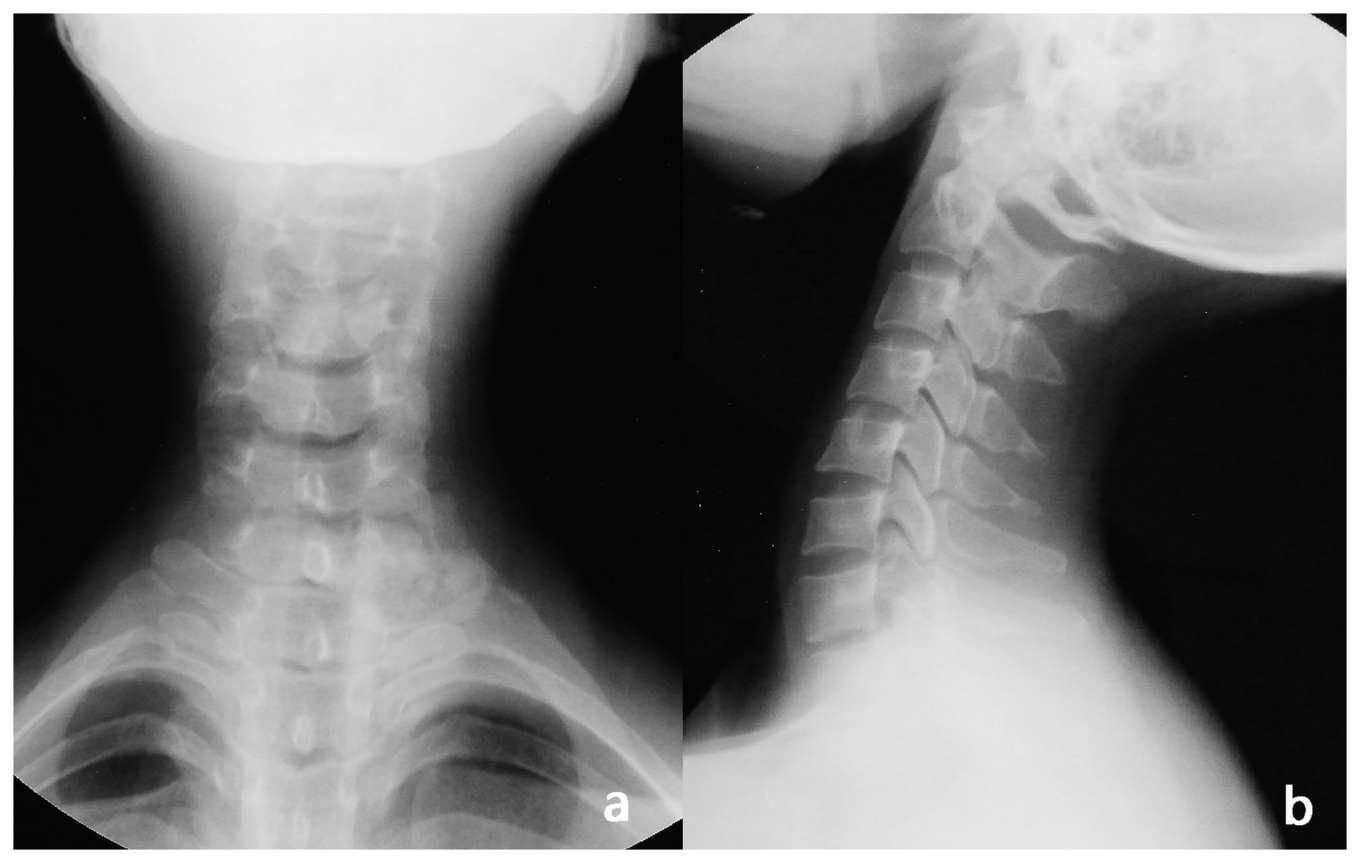
Anteroposterior and lateral radiographs of the cervical vertebrae of a 33year-old female. a: anteroposterior radiograph; b: lateral radiograph. The patient was admitted to our hospital with primary complaints of neck and back pain for 4 years, with pain radiating to the right upper arm and numbness in right hand for 3 years.

**Figure 2:**
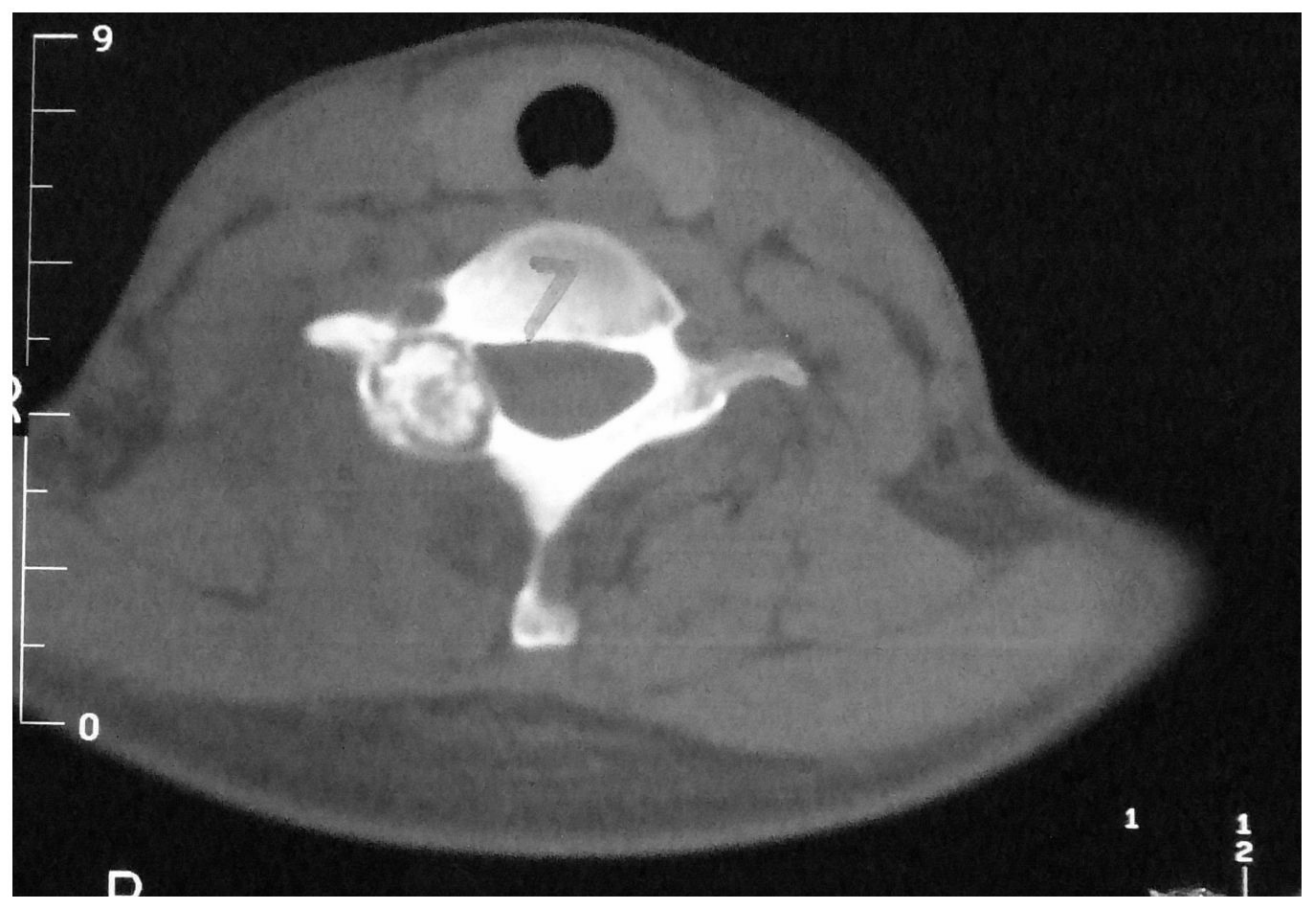
Preoperative CT scan shows a lesion located at the posterior elements of the C7 vertebra. There is an expansile lesion with cortical destruction and surrounding sclerosis. The boundaries are unclear and mottled densities of calcification can be seen within the lesion.

**Figure 3:**
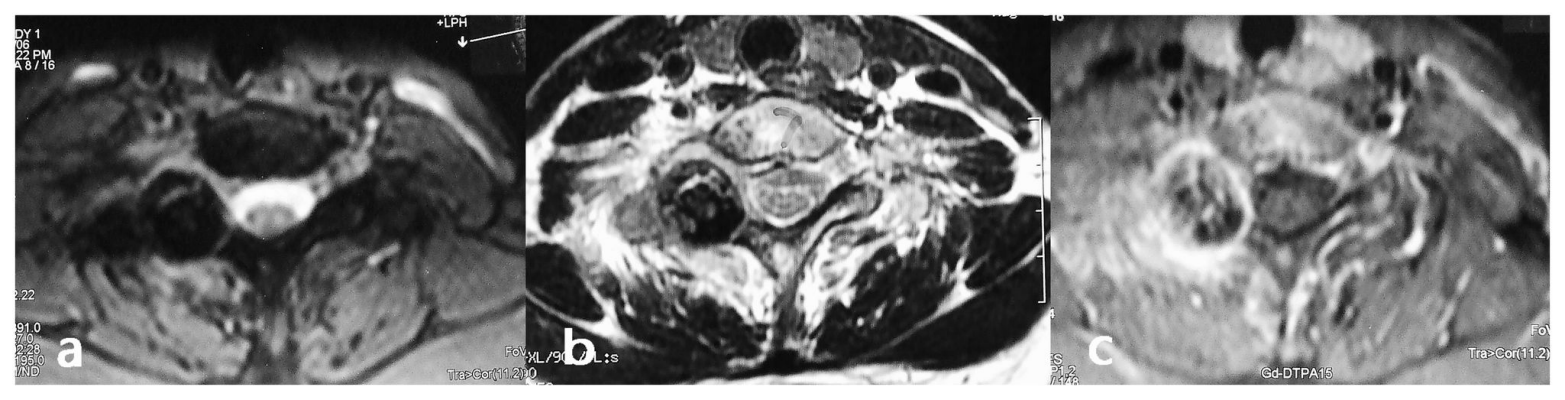
Preoperative MRI demonstrates single cystic lesion with cortical destruction at the posterior elements of C7 vertebra and slight edema of the surrounding soft tissues. Enhanced MRI shows a rich vascular supply to osteoid tissue, and the image has been enhanced significantly. a,b: T1 and T2 weighted image; c: enhanced MRI.

**Figure 4:**
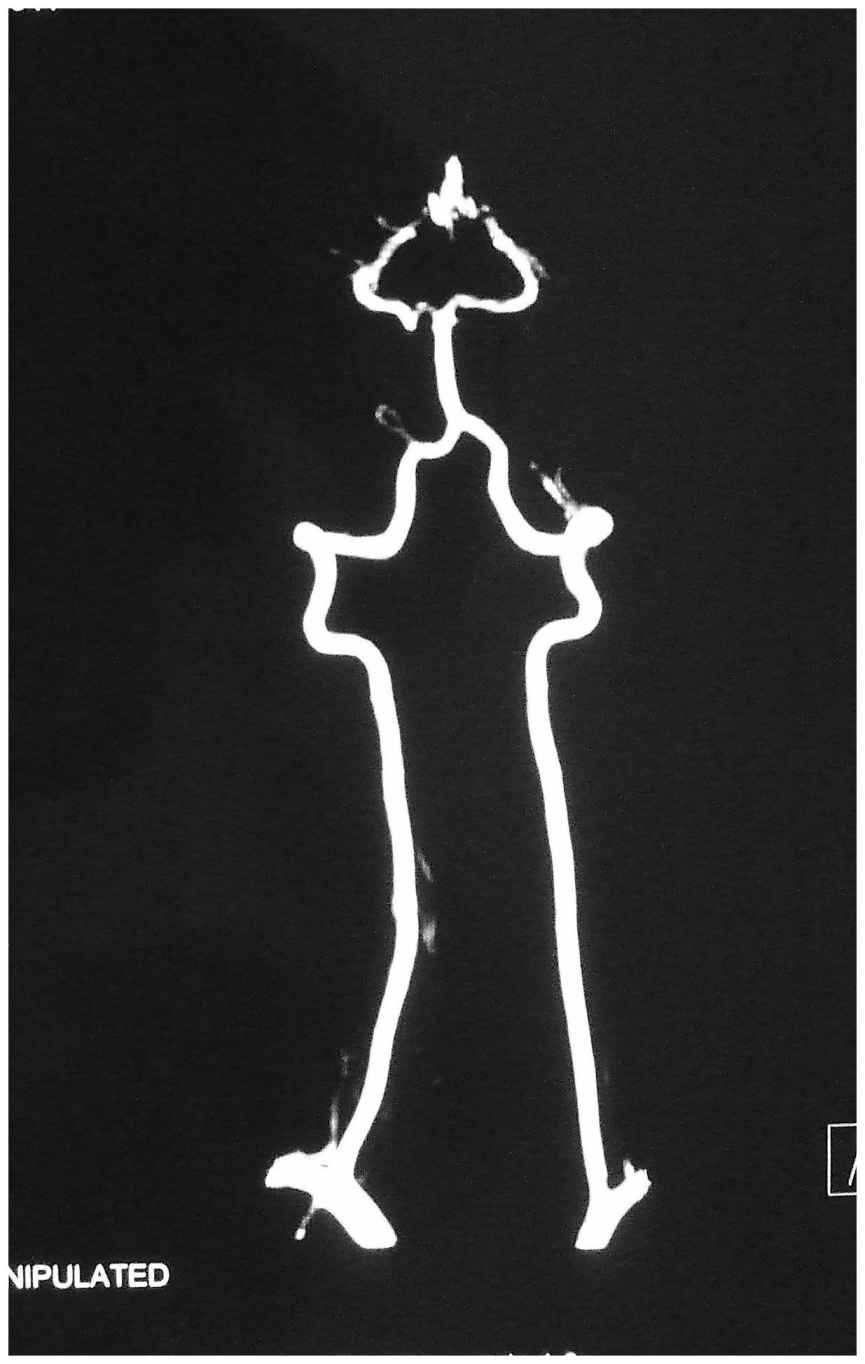
Preoperative MRA reveals no abnormalities of the cervical arteries.

**Figure 5:**
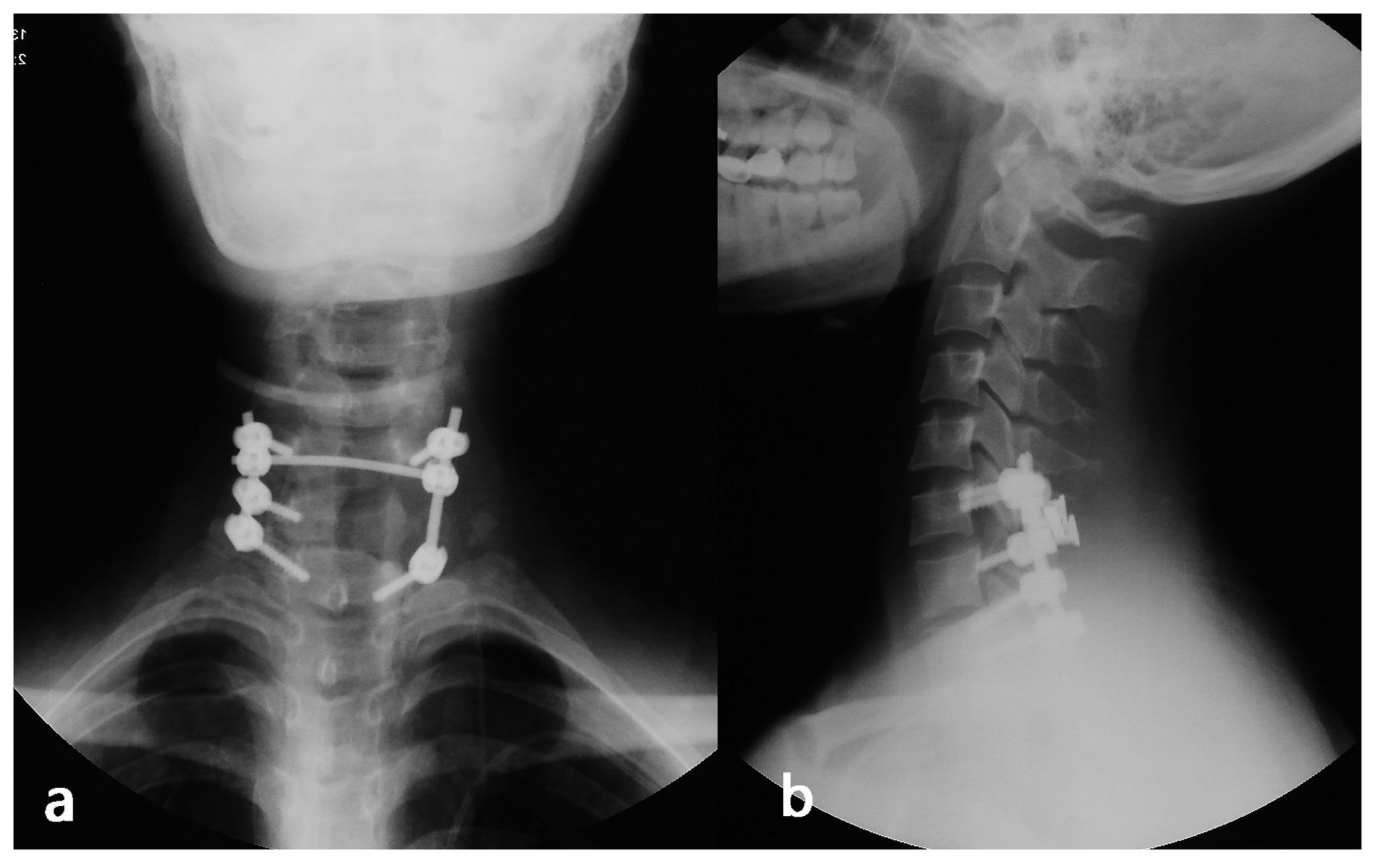
Postoperative AP and lateral radiographs of cervical vertebrae. a: anteroposterior radiograph; b: lateral radiograph. The patient underwent posterior cervical en bloc resection of the tumor, spinal cord decompression, pedicle screw internal fixation as well as spinal fusion using a bone graft.

**Figure 6:**
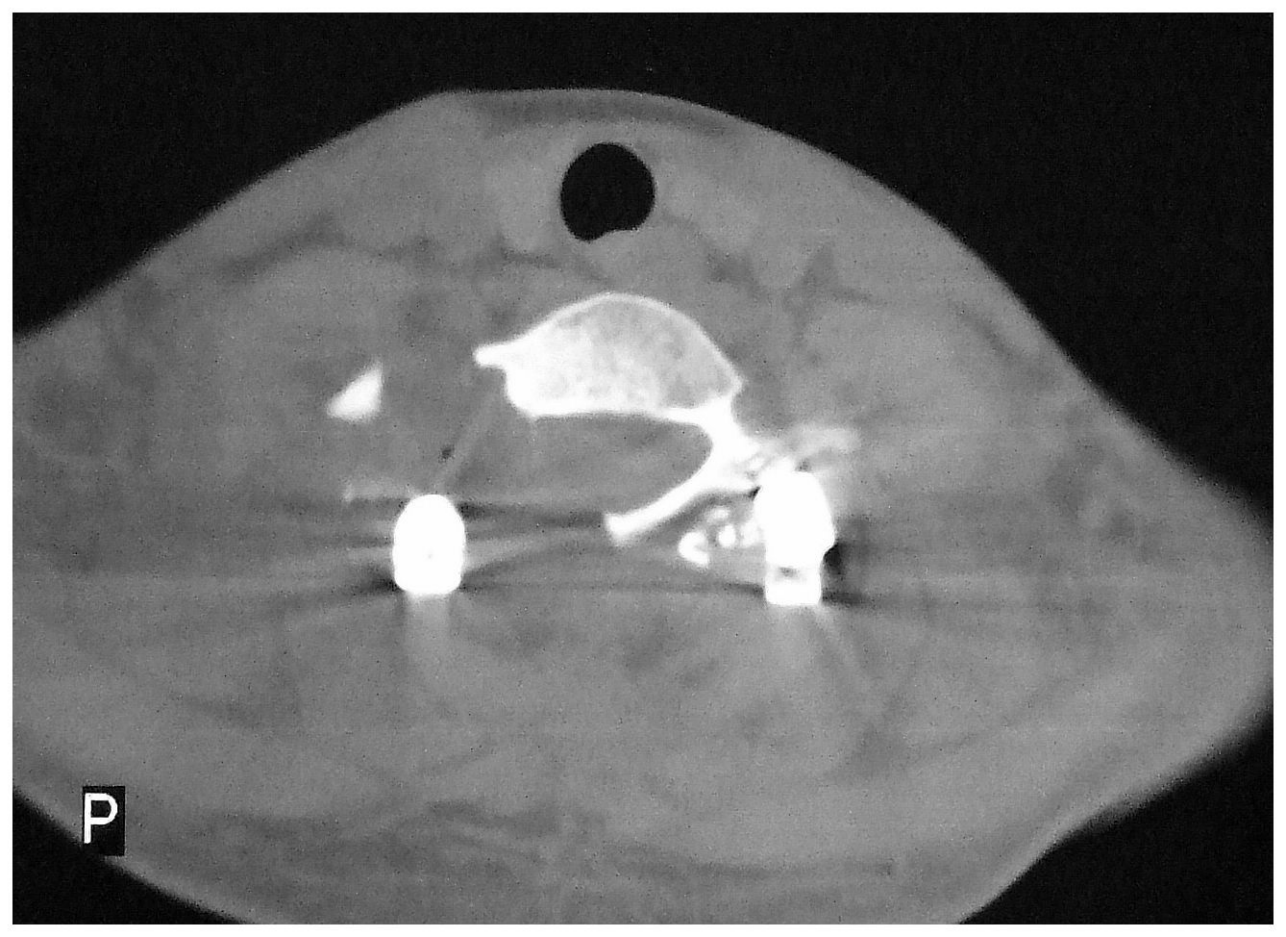
Postoperative CT scan showing the extents of en bloc resection of the tumor and decompression.

**Figure 7:**
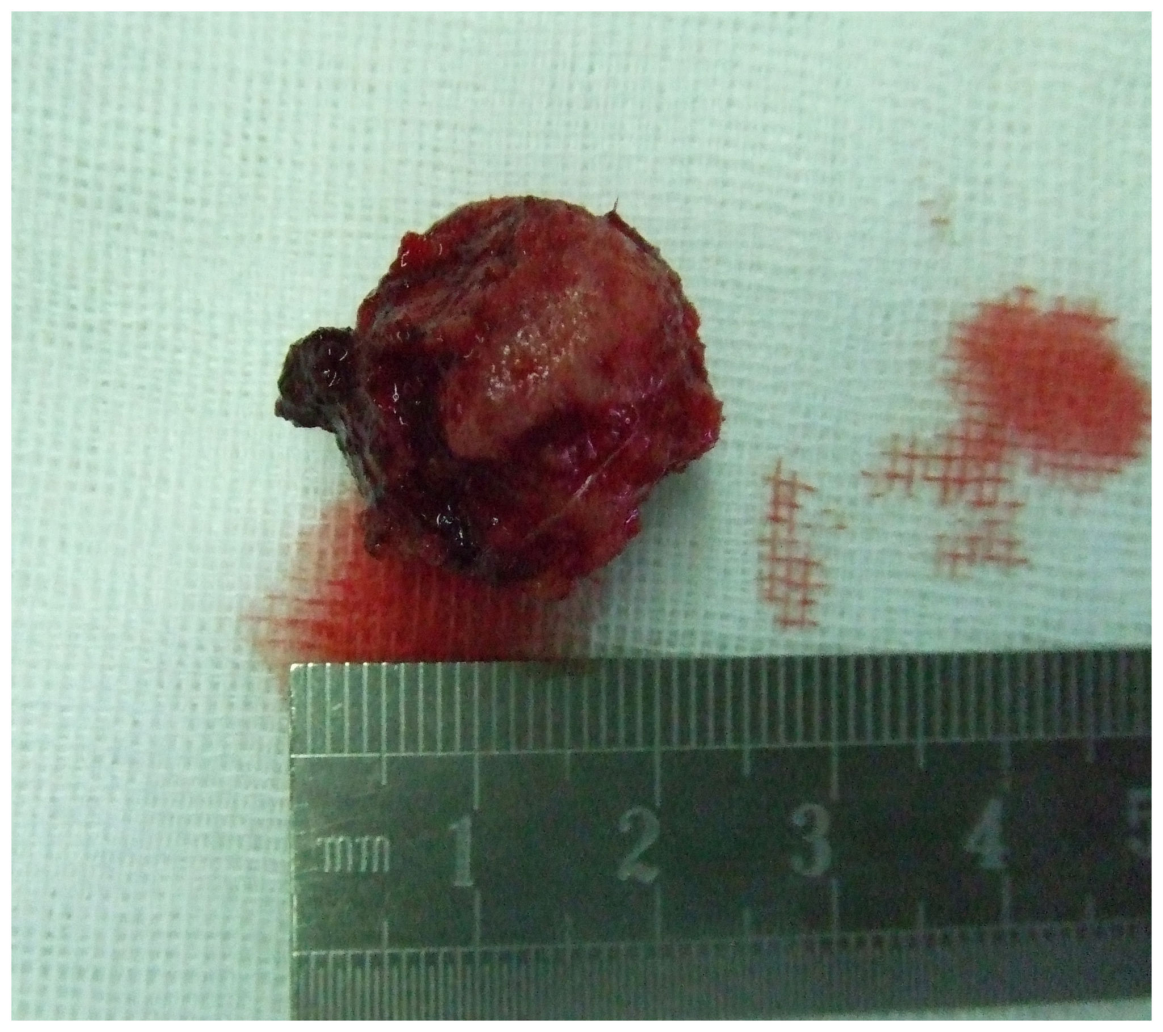
En bloc excised tumor tissue.

**Figure 8:**
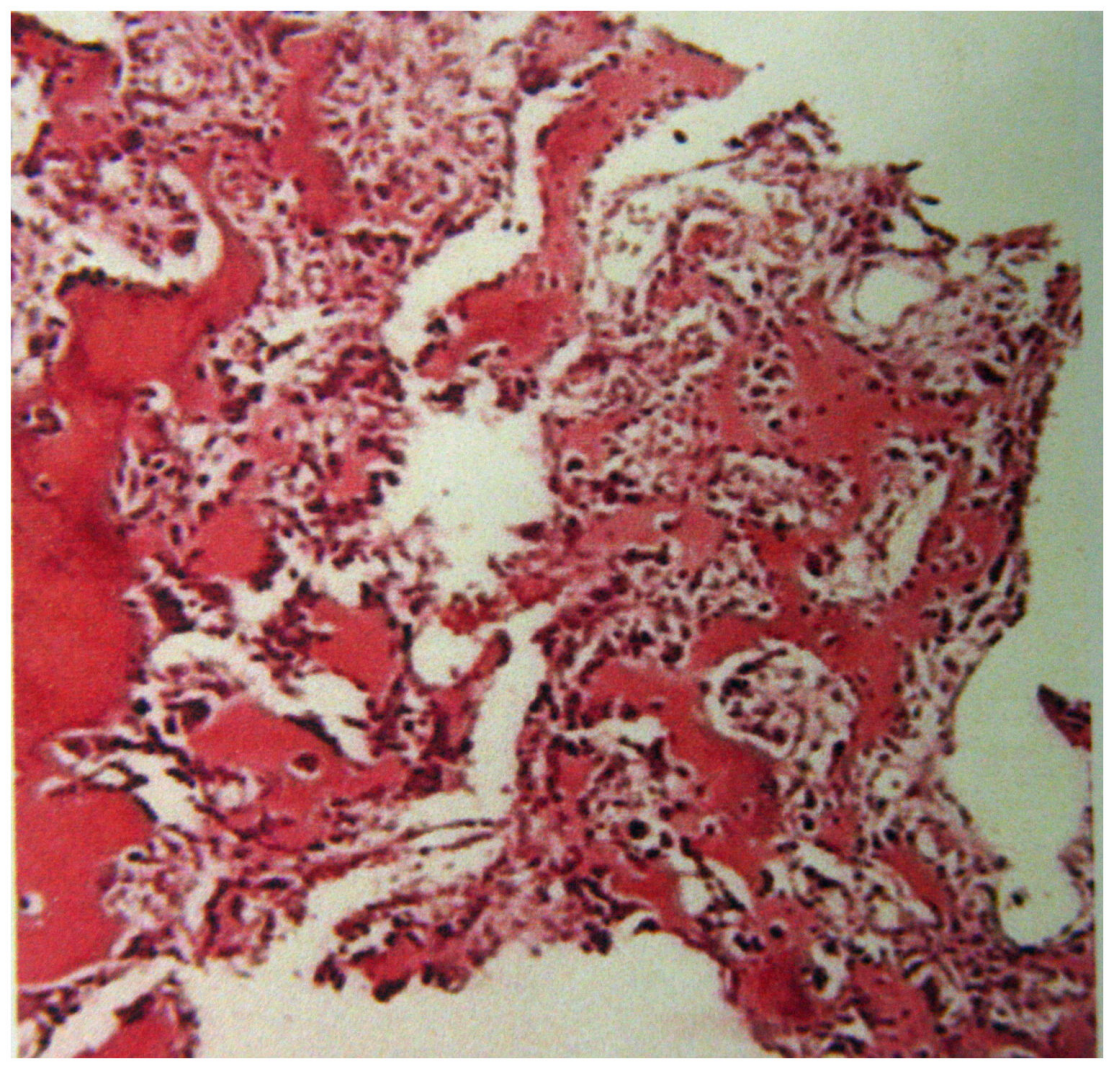
Pathological findings reveal that the tumor stroma contains mature osteoblasts, which are round or oval in shape and have a rich cytoplasm. No nuclear atypia are present. The tumor consists of anastomosing trabeculae of woven bone produced by osteoblasts, and shows deposition of calcium salt.

The follow-up period was from 24 to 80 months (average 38.4 months) for 18 patients in this study. The operative duration ranged between 80 and 220 minutes (average 120.8 minutes). The intraoperative bleeding volume ranged between 300 and 1200 ml (average 520 ml). There was no incidence of intraoperative complications, including worsening of spinal cord or nerve root injury, vertebral artery damage or tearing of spinal dura matter. In this study, complete excision of the tumor was achieved in all the patients, and various extents of bone destruction and thinning at the lesion site could be observed. The nidus of the tumor was over 1.5 cm in diameter. Gross examination showed clear boundaries and purple-red cut surface of the lesion. The specimen was friable and tended to bleed. All the specimens were sent for histopathology assessment and revealed that the tumor was composed of large amount of osteoid tissue, containing numerous osteoblasts. The osteoblasts were round or oval in shape, and were well differentiated, without any cellular atypia. The preoperative and final follow-up VAS scores were 6.46±1.32 and 2.26±1.05, respectively, which was significantly decreased (*p*<0.05). According to McCormick classification, the preoperative spinal cord function was classified as grade I in 4 patients, grade II in 11 patients, grade III in 2 patients and grade IV in 1 patient, and the final follow-up spinal cord function was classified as grade I in 15 patients, grade II in 2 patients and grade III in 1 patients (Table 1). Spinal cord function was improved in 13 patients and did not change in 5 patients at final follow-up visit compared to the preoperative function. No recurrence of tumors, loosening, migration or rupture of bone graft were discovered in any patients during the follow-up period (Figures 1-8).

**Table 1:** Preoperative and final follow-up spinal cord functional grades according to McCormick classification.

**McCormick classification**	**Preoperative**	**Final follow-up**
Grade I	4	15
Grade II	11	2
Grade III	2	1
Grade IV	1	0

## Discussion

### Clinical features and pathological changes

Osteoblastoma is an uncommon benign primary bone tumor arising from osteoblasts and osteoid tissues. The incidence of osteoblastoma is low and the male to female patient ratio is 2:1, and predominantly affects individuals under the age of 30. It is usually present in the vertebral column and long bones, followed by the bones of the hands and feet; however, the mandible and tissues other than bone have also been reported in the literature as sites of osteoblastoma. The spinal lesions usually involve the transverse process and spinous process of the vertebrae. Although the vertebral bodies can be involved, it is extremely rare to find a tumor primarily located in the vertebral bodies [1-4].

In this study, there were 15 patients under the age of 30 and the mean age was 27.5 years. The male to female ratio was 1.57:1. The tumors involved the posterior elements of the spine in 16 patients and vertebral bodies in 2 patients. The sites of osteoblastoma and the mean age of patients in this study were both consistent with those reported in the literature. According to previous literatures, there was no difference for the incidence of osteoblastoma in the location of cervical spine, thoracic spine and lumbar spine. However it was showed that the tumors were majorly located at the cervical vertebrae and the cervicothoracic junction in 17 out of 18 patients in this retrospective study. The high incidence of osteoblastoma in the cervical vertebrae in this study may be biased due to the small sample size or biological behavior of osteoblastoma; however this phenomenon remained to be further investigated in further studies.

The clinical presentation of osteoblastoma differs depending on the tumor site. Patients with a tumor present in the extremities often experience local dull pain that does not worsen at night. Local soft tissue edema or a mass are sometimes detected in these patients. Patients with the tumor located near the joints will suffer from limitation of limb movements. Local pain is the most common symptom of spinal osteoblastoma. Scoliosis and myelopathic and/or radicular symptoms may also be detected. All patients in this study experienced various intensities of posterior neck pain or back pain, with some patients complaining of night pain. Radicular pain occurred in 10 patients, sensory disorder was experienced by 7 patients, various intensities of dyskinesia below the tumor affecting vertebral segments were discovered in 8 patients, and sphincter dysfunction was found in 2 patients.

The gross appearances of the tumors varied in terms of pathological findings. The tumor nidus in these cases was similar to that of osteoid osteoma, with a diameter of over 1.5 cm. The tumors were commonly purple-red in color, solid, and had the texture of a pebble. These tumors also had a rich vascular supply. Foci of hemorrhage, cystic change, or granulation tissue formation, and secondary bone cysts could be observed in some specimens. Microscopically, extensive or trabeculated osteoid formation was observed, and various degrees of focal calcification or ossification was seen in the tumor tissue. Numerous hyperplastic osteoblasts were present in the bone trabeculae with highly vascular stroma. A small number of polykaryocytes, osteoclasts and lymphocytes were also present. The neoplastic cells generally showed no significant cellular atypia or nuclear division [12].

Studies show that some osteoblastomas contain cells with a degree of cellular atypia and are biologically aggressive carcinomas. These tumors are generally associated with recurrence and malignant transformation when they are not excised completely. Therefore, osteoblastomas have been divided into two subgroups: benign and malignant (aggressive) [13,14]. The pathological findings of the specimens from our study indicated benign tumors in 18 patients.

### Diagnosis and differential diagnosis

The diagnosis of spinal osteoblastoma is reliant on the results from imaging and pathological examinations due to the lack of a specific clinical manifestation. The tumor generally arises within the posterior spinal elements and the characteristic appearance of osteoblastoma on X-rays is a cystic, expansile, well-defined lesion of low signal intensity, with cortical thinning or rupture and a distinct egg-shell calcification around the focus. The presence of scattered or extensive foci of calcification or ossification within the lesion can be very helpful in diagnosis.

Computed tomography (CT) scans can provide the information about the location, extent, size and borders of the lesion in bone. It is best for the observation of the fine structure of the tumor, the intensity of cortical destruction and the formation of a bone shell, as well as whether there exists soft tissue masses. The characteristic CT appearance of spinal osteoblastoma includes the following: the lesions are located at the posterior elements; the expansile lesion destroys the normal bone tissue; there is surrounding sclerosis lacking clear boundaries; there might be soft tissue masses presented; and mottled densities from calcification can be observed within the lesion. CT scans are also useful in detecting the relationship between the lesion and the surrounding tissues, especially in determining whether there is spinal canal stenosis or thecal sac compression. In addition, CT is obviously superior to MRI in demonstrating the scattered or extensive foci of calcification or ossification in some lesions [14,15].

Combined with clinical assessment, MRI plays an important role in the qualitative diagnosis, preoperative localization and staging of osteoblastomas. MRI is particularly valuable in displaying secondary spinal cord changes, which is important for choosing a treatment method and defining a prognosis. However, the appearances of bone and soft tissue tumors are generally nonspecific with MRI. This test is not able to distinguish the punctate foci of calcification or ossification, and is inferior to a CT scan in the demonstration of cortical destruction. The number of cortical destruction sites and the anatomical relationship between the tumor and the surrounding bone tissues are better shown by CT scan. The appearance of tumors via MRI is usually similar to that of other types of bone tumors. An osteoblastoma often shows isointensity or hypointensity on T1-weighted images (T1WI), and heterogeneously hypointensity and hyperintensity on T2-weighted images (T2WI). Solitary or multiple bone rupture due to cystic expansile lesions can be discovered and sometimes fluid-fluid levels can be seen. Slight edema-type changes in the adjacent tissues might be found while soft tissue masses usually cannot be shown clearly. Contrast enhanced MRI will display a rich vascular supply in the osteoid tissue with greatly enhanced signal intensity, while the signal intensity representing calcification or ossification, cystic change and bleeding will not be enhanced [14,16].

Final diagnosis of spinal osteoblastoma cannot be made based only on medical history, physical examination and imaging test findings. Pathological evaluation of the lesion should be included for a definitive diagnosis. In order to be definitively classified, spinal osteoblastoma needs to be differentiated from the following benign tumors or tumors of low malignant potential: 1) Osteoid osteoma: The histologic manifestation of osteoblastoma is similar to that of osteoid osteoma. These two types of tumors differ mainly in size, features of pain, and the existence of a rim of reactive bone around the tumor nidus. The pain associated with osteoid osteoma is usually typical, with a significant increase of intensity at night, and can be relieved readily with salicylates. The extents of bone destruction from osteoid osteomas tend to be less than 2 cm in diameter with no obvious spansile lesions. Reactive bony sclerosis around the nidus can be observed, and cortical thickening as well as periosteal reaction may be demonstrated around the area of bone destruction. 2) Giant cell tumor of bone (GCTOB): This type of tumor generally occurs in male adults in the third and fourth decades of life. The lesion is most often eccentrically located or shows horizontal extension. In imaging findings, these tumors usually show the characteristic “soap bubble” appearance. Occasionally, mixed lytic and sclerotic changes might be displayed. Various extents of lytic changes and residual bone trabeculae are present within the lesion with generally no punctate foci of calcification or sclerotic borders. It is rare to find periosteal reaction or fluid-fluid levels. In this type of tumor, there will be no obvious enhancement of the signal intensity. In contrast to GCTOB, osteoblastomas are usually present with foci of calcification or ossification. Some have thick sclerotic borders, sometimes existing as a node shape. Images of the lesion can be greatly enhanced with contrasts. 3) Aneurysmal bone cyst (ABC): This type of bone cyst usually affects young adults under the age of 30 and is slightly more common in males. ABCs are divided into two types: primary and secondary cysts. X-rays of ABCs characteristically demonstrate an expansile osteolytic lesion with various thicknesses of trabeculae, showing a honeycomb appearance. The lesion is well-defined and surrounded by an intact shell of sclerotic bone. When an aneurysmal bone cyst arises from a pre-existing osteoblastoma and the two lesions are hard to differentiate, a relatively specific appearance of foci of calcification or ossification within the osteoblastoma will be helpful in making a diagnosis. 4) Metastatic spinal tumors: These patients usually have a history of primary tumors. They are generally found in middle-aged and older adults. Imaging tests show these metastatic tumors are located at the posterolateral epidural space. The vertebral bodies are obviously destructed and can even lead to vertebral collapse, while intervertebral discs are usually intact. These lesions commonly involve multiple vertebral bodies and posterior elements and are demonstrated as skip lesions. There will be distinct soft tissue masses and the intensity of the lesion will be enhanced by contrast. 5) Spinal tuberculosis: The patients that are usually affected by spinal tuberculosis are young adults that often have the history of tuberculosis at other sites and always have a positive tuberculosis skin test. The imaging test findings include: destruction of adjacent vertebral bodies and the intervertebral disc; intervertebral space stenosis; sclerotic borders around the destructed bone tissue; and accompanying paravertebral abscess with a signal intensity that is not enhanced by contrast.

Schajowicz et al described a malignant or aggressive type of osteoblastoma in 1976 [14]. This type of osteoblastoma is considered to be a borderline (or low grade malignant) osteoblastic tumor with intermediate biological behavior between that of benign osteoblastoma and that of osteosarcoma. This type of osteoblastoma accounts for 0.28% of all primary bone tumors. Aggressive osteoblastoma can evolve from osteoid osteoma or benign osteoblastoma. It can also be an aggressive from the very beginning of its development, and some will evolve into osteosarcoma. Clinicians should be aware of the potential risks of malignant transformation and make a diagnosis quickly when the following clinical and imaging demonstrations are present in patients with osteoblastoma: intense pain which is worse at night with significant local tenderness; imaging tests showing calcified foci within soft tissue masses, faintly visible expanded areas of bone destruction, periosteal reaction, foci of calcification within the lesion, and decreased ossification; and recurrence of the tumor.

### Treatment and surgical precautions

Especially aggressive osteoblastomas tend to recur and may evolve into osteosarcomas. The tendency to recur depends on tumor type and treatment method. It has been reported that the recurrence rate for osteoblastoma is 12.5-50% when excochleation is adopted. Multiple recurrence and malignant transformation of benign osteoblastoma have also been reported in the literature [13,14]. At present, the most effective treatment for spinal osteoblastoma is en bloc resection of the tumor, which will suppress recurrence to a certain extent. Spinal decompression therapy needs to be adopted when spinal cord or nerve root compression is present. When a definite diagnosis is made, complete tumor removal should be performed as early as possible while at the same time, further injury to the spinal cord or nerve root should be prevented [12,17]. If the tumor is an aggressive type, adjuvant chemotherapy and radiotherapy should be used after surgery [18].

Several precautions should be taken into consideration while performing surgery for spinal osteoblastoma. (1) First and foremost, the tumor should be sufficiently separated from the adjacent tissues and completely excised. Although it is ideal to sufficiently separate the tumor to help determine the extent of tumor excision, the procedure is difficult to perform when the tumor is located at complex anatomical sites, is extensive, or when the exposure of the lesion is limited. Therefore, piecemeal resection has to be performed to achieve the necessary extensive range of resection. For cases of osteoblastomas arising within the posterior elements and extending to the vertebral bodies, piecemeal resection through a combined anterior and posterior approach followed by spinal reconstruction for stability can be used when the tumors are located at cervical vertebrae, thoracic vertebrae or the thoracolumbar junction, while the single posterior approach can be adopted when the tumors are located at the lumbar vertebrae.

(2) Intraoperative hemostasis should also be taken into account when considering precautions for spinal osteoblastoma surgery. Preoperative magnetic resonance angiography (MRA) or digital subtraction angiography (DSA) should be routinely performed to assess bilateral vertebral arteries if the anatomical relationship between the vertebral arteries and the lesion is unclear. Preoperative MRA of the cervical arteries was performed in 6 patients in this study. Vertebral artery compression on the affected side was found in 4 patients and compensatory enlargement of the vertebral artery on the unaffected side was observed in 2 of the 4 patients. Careful observation of the relationship between the tumor and the vertebral artery is needed before separation during the surgery. The vertebral artery is surrounded by a very rich venous plexus, and hemostasis cannot be achieved easily when bleeding occurs, which will always result in incomplete resection of the tumor. Therefore, when venous plexus bleeding is encountered, electrocautery can be used to achieve hemostasis when the bleeding occurs within the direct field-of-view, and gelatin sponges or neuropatties should be used for compression hemostasis when the bleeding is outside of the direct field-of-view. Inappropriate bipolar coagulation for hemostasis should be avoided; otherwise the adjacent tissues might be injured.

(3) Sufficient decompression must be obtained in order to expose the maximal portion of the tumor. This will lower the risk of intraoperative spinal cord injury. Sometimes decompression needs to be achieved at the expense of normal bone structure. The electric current for bipolar coagulation and the suction force of the suction device need to be reduced to avoid spinal cord injury secondary to traction, overheating, and excessive suction. Intraoperative traction of the spinal cord should be avoided since even simple traction may result in irreversible spinal cord injury. The spinal cord should always be protected during the procedure of spinal tumor resection, and when necessary, piecemeal resection of the tumor shall be considered.

(4) Neurophysiological monitoring is an essential precaution to take during spinal surgery. Neurophysiological monitoring such as SSEP or motor-evoked potentials (MEP) is sometimes performed during spine surgery to assess the function of the spinal cord or nerve root and to prevent iatrogenic injury. SSEPs have been utilized as an intraoperative monitoring tool for over 30 years [19]. They are currently used either to assess the functional status of somatosensory pathways during surgical procedures which may affect peripheral nerve or plexus, spinal cord, brainstem, and brain function or to identify the sensory portion of the sensorimotor cortex. When used to assess function, SSEP responses are typically elicited by stimulation at a peripheral site distal to the structure at risk and may be recorded at a distal site and one or more sites proximal to the structure at risk. The distal recording site is used to insure effective stimulation and the proximal recording sites are used to monitor the changes that may occur with functional compromise of the structure in question [19,20]. MEPs monitor corticospinal track activity via stimulation at the level of the motor cortex or spinal cord and are selective for motor pathways [21]. MEP monitoring provides a repeatable snap shot that permits immediate assessment of spinal cord function after high-risk maneuvers such as correction. MEP monitoring is linked to few untoward effects and has clear advantages but still requires that other monitoring methods such as SSEPs be used to provide optimal information [21,22]. In our research SSEPs were utilized as an intraoperative monitoring tool for all the cases, iatrogenic spinal cord injury was effectively avoided.

(5) Spinal reconstruction for stability is also essential for surgeries for osteoblastoma of the spine. Spinal stability mainly depends on muscles, vertebrae, intervertebral discs and fibrous rings. Other structures such as the anterior and posterior longitudinal ligaments and facet joints also play an important role in maintaining the stability of spine. The spinal column will lose stability when any of the spinal structures mentioned above are destructed. Without appropriate fixation and support, spinal cord compression will develop due to spinal malformation or dislocation of a vertebra, which will result in severe and potentially life-threatening consequences.

In 13 patients in this study, the lesions involved facet joints, pedicles and anterior vertebral bodies. Spinal stability was affected severely by tumor resection procedure and spinal reconstruction was performed to restore the stability. The posterior elements and anterior vertebral bodies were both involved in 2 out of the 13 patients, and anterior spinal fusion using a bone graft and internal metal plate fixation were performed after tumor resection. For the remaining 11 patients, posterior vertebral pedicle or lateral mass fixation with screws was performed. The tumor was only affecting the lamina and the spinal stability was not affected in the other 5 patients, therefore, only tumor excision was performed. Postoperative bracing was required for protection in all patients for 3 months. Follow-up X-rays showed that the spinal stability was restored and no kyphosis occurred. Spinal fusion was achieved within 3 to 6 months.

## References

[1] Lucas DR (2010) Osteoblastoma. Arch Pathol Lab Med 134: 1460-1466. PubMed: 20923301. 20923301 10.5858/2010-0201-CR.1

[2] Greenspan A (1993) Benign bone-forming lesions: osteoma, osteoid osteoma, and osteoblastoma. Clinical, imaging, pathologic, and differential considerations. Skeletal Radiol, 22: 485-500. PubMed: 8272884. 8272884 10.1007/BF00209095

[3] Ropper AE, Cahill KS, Hanna JW, McCarthy EF, Gokaslan ZL et al. (2011) Primary vertebral tumors: a review of epidemiologic, histological, and imaging findings, Part I: benign tumors. Neurosurgery,69(6): 1171-1180. doi:10.1227/NEU.0b013e31822b8107. PubMed: 21725252. 21725252 10.1227/NEU.0b013e31822b8107

[4] Lee EJ, Park CS, Song SY, Park NH, Kim MS (2004) Osteoblastoma arising from the ethmoidal sinus. AJR Am J Roentgenol, 182: 1343-1344. doi:10.2214/ajr.182.5.1821343. PubMed: 15868703. 15868703 10.2214/ajr.182.5.1821343

[5] Boriani S, Amendola L, Bandiera S, Simoes CE, Alberghini M et al. (2012) Staging and treatment of osteoblastoma in the mobile spine: a review of 51 cases. Eur Spine J, 21: 2003-2010. doi:10.1007/s00586-012-2395-8. PubMed: 22695702. 22695702 10.1007/s00586-012-2395-8 PMC3463681

[6] Ramme AJ, Smucker JD (2011) Balancing spinal stability and future mobility in the cervical spine: surgical treatment of a case of osteoblastoma with secondary aneurysmal bone cyst. Spine J, 11(5): e5-12. doi:10.1016/j.spinee.2011.03.012. PubMed: 21558033. 10.1016/j.spinee.2011.03.012 21558033

[7] Kan P, Schmidt MH (2008) Osteoid osteoma and osteoblastoma of the spine. Neurosurg Clin N Am,19(1): 65-70. doi:10.1016/j.nec.2007.09.003. PubMed: 18156049. 18156049 10.1016/j.nec.2007.09.003

[8] Poleksić ZR, Lalosević VJ, Milinković ZB (2010) Osteoblastoma of the spine. Acta Chir Iugosl, 57(1): 63-68. doi:10.2298/ACI1001063P. PubMed: 20681202. 20681202 10.2298/aci1001063p

[9] Amirjamshidi A, Abbassioun K (2010) Osteoblastoma of the third cervical vertebra in a 16-year-old boy: case report and review of the literature. Pediatr Neurosurg, 46: 396-401. doi:10.1159/000323422. PubMed: 21412027. 21412027 10.1159/000323422

[10] Boriani S, Weinstein JN, Biagini R (1997) Primary bone tumors of the spine terminology and surgical staging. Spine, 22: 1036-1044. doi:10.1097/00007632-199705010-00020. PubMed: 9152458. 9152458 10.1097/00007632-199705010-00020

[11] McCormick PC, Torres R, Post KD, Stein BM (1990) Intramedullary ependymoma of the spinal cord. J Neurosurg, 72: 523-532. doi:10.3171/jns.1990.72.4.0523. PubMed: 2319309. 2319309 10.3171/jns.1990.72.4.0523

[12] Hart RA, Boriani S, Biagini R, Currier B, Weinstein JN (1997) A system for surgical staging and management of spine tumors. A clinical outcome study of giant cell tumors of the spine. Spine, 22: 1773-1782. doi:10.1097/00007632-199708010-00018. PubMed: 9259790. 9259790 10.1097/00007632-199708010-00018

[13] Samdani A, Torre-Healy A, Chou D, Cahill AM, Storm PB (2009) Treatment of osteoblastoma at C7: a multidisciplinary approach. A case report and review of the literature. Eur Spine J,18 Suppl 2: 196-200. doi:10.1007/s00586-008-0806-7. 10.1007/s00586-008-0806-7 PMC2899567 18839223

[14] Schajowicz F, Lemos C (1976) Malignant osteoblastoma. J Bone Joint Surg Br, 58: 202-211. PubMed: 932083. 932083 10.1302/0301-620X.58B2.932083

[15] Okuda S, Myoui A, Nakase T, Wada E, Yonenobu K et al. 1 (2001) Ossification of the ligamentum flavum associated with osteoblastoma: a report of three cases. Skeletal Radiol,30: 402-406 10.1007/s002560100324 11499782

[16] Gualdi GF, Casciani E, Di Biasi C, Trasimeni G, Postacchini F (1999) The role of CT and MRI in the identification, characterization and staging of tumors of the spinal vertebrae. Clin Ter,150:51-65 10367545

[17] Lian X, Zhao J, Hou T, Ma H, Chen Z (2006) Benign intraspinal osteoblastoma stemming from C7 lamina in cervicothoracic junction: a case report. Spine 31: E895-E899. doi:10.1097/01.brs.0000244666.91119.a1. PubMed: 17077728. 17077728 10.1097/01.brs.0000244666.91119.a1

[18] Batay F, Savaş A, Uğur HC, Kanpolat Y, Kuzu I (1998) Benign osteoblastoma of the orbital part of the frontal bone: case report. Acta Neurochir, 140: 729-730. doi:10.1007/s007010050172. PubMed: 9781291. 9781291 10.1007/s007010050172

[19] Nash CL Jr, Lorig RA, Schatzinger LA, Brown RH (1977) Spinal cordmonitoring during operative treatment of the spine. Clin Orthop, 126: 100-105. PubMed: 598095. 598095

[20] Sand T, Kvaløy MB, Wader T, Hovdal H (2013) Evoked potential tests in clinical diagnosis. Tidsskr nor Laegeforen 133: 960-965. doi:10.4045/tidsskr.12.1176. PubMed: 23652144. 23652144 10.4045/tidsskr.12.1176

[21] Malhotra NR, Shaffrey CI (2010) Intraoperative electrophysiological monitoring in spine surgery. Spine, 35: 2167-2179. doi:10.1097/BRS.0b013e3181f6f0d0. PubMed: 21102290. 21102290 10.1097/BRS.0b013e3181f6f0d0

[22] Sloan TB, Janik D, Jameson L (2008) Multimodality monitoring of the central nervous system using motor-evoked potentials. Curr Opin Anaesthesiol,21: 560-564. doi:10.1097/ACO.0b013e32830f1fbd. PubMed: 18784479. 18784479 10.1097/ACO.0b013e32830f1fbd

